# Taheebo Polyphenols Attenuate Free Fatty Acid-Induced Inflammation in Murine and Human Macrophage Cell Lines As Inhibitor of Cyclooxygenase-2

**DOI:** 10.3389/fnut.2017.00063

**Published:** 2017-12-12

**Authors:** Sihui Ma, Koichi Yada, Hyunjin Lee, Youichi Fukuda, Akira Iida, Katsuhiko Suzuki

**Affiliations:** ^1^Graduate School of Sport Sciences, Waseda University, Tokorozawa, Japan; ^2^Faculty of Sport Sciences, Waseda University, Tokorozawa, Japan; ^3^Research Organization for Nano and Life Innovation, Waseda University, Tokorozawa, Japan; ^4^Faculty of Agriculture, Kindai University, Nakamachi, Japan

**Keywords:** polyphenols, anti-inflammatory agents, cyclooxygenase-2, RAW264.7 cells, THP-1 cells

## Abstract

**Aim of study:**

Taheebo polyphenols (TP) are water extracts of *Tabebuia* spp. (Bignoniaceae), taken from the inner bark of the *Tabebuia avellanedae* tree, used extensively as folk medicine in Central and South America. Some anti-inflammatory drugs act by inhibiting both cyclooxygenase-2 (COX-2) and COX-1 enzymes. COX-2 syntheses prostaglandin (PG) E_2_, which is a species of endogenous pain-producing substance, whereas COX-1 acts as a house-keeping enzyme. Inhibiting both COX-1 and -2 simultaneously can have side effects such as gastrointestinal bleeding and renal dysfunction. Some polyphenols have been reported for its selective inhibiting activity toward COX-2 expression. Our study aimed to demonstrate the potential and mechanisms of TP as an anti-inflammation action without the side effects of COX-1 inhibition.

**Materials and methods:**

Free fatty acid-stimulated macrophage cell lines were employed to mimic macrophage behaviors during lifestyle-related diseases such as atherosclerosis and non-alcoholic steatohepatitis. Real-time polymerase chain reaction was used to detect expression of inflammatory cytokine mRNA. Griess assay was used to measure the production of nitric oxide (NO). ELISA was used to measure PG E_2_ production. Molecular docking was adopted to analyze the interactions between compounds from *T. avellanedae* and COX-2.

**Results:**

TP significantly suppressed the production of NO production, blocked the mRNA expression of iNOS, and COX-2 in both cell lines, blocked the mRNA expression of TNF-α, IL-1β, IL-6, and PGE_2_ in the murine cell line. However, there was no inhibitory effect on COX-1. Molecular docking result indicated that the inhibitory effects of TP on COX-2 and PGE_2_ could be attributed to acteoside, which is the main compound of TP that could bind to the catalytic zone of COX-2. After the interaction, catalytic ability of COX-2 is possibly inhibited, followed by which PGE_2_ production is attenuated. COX inhibitor screening assay showed TP as a selective inhibitor of COX-2 enzyme.

**Conclusion:**

The anti-inflammatory effects of TP can possibly regulate macrophages due to the targeted inhibition of COX-2 activity, without affecting COX-1 activity with other anti-inflammatory effects including suppression of iNOS and inflammatory cytokines. As such, TP is potentially useful in prevention and treatment of lifestyle-related disease by attenuating inflammation caused by macrophages infiltration.

## Introduction

Screening bioactive substances with novel structures and unique pharmacological activities from natural products is an efficient method for research and development in new drugs and dietary-sourced supplements. According to the Food and Drug Administration of the USA, during 1983–2010, over 1,000 kinds of small-molecule substances were approved to be used clinically; 50% of which originated from natural products (1). Historically, many drugs discovered from natural products, such as the most famous one, aspirin, firstly isolated from willow bark as the compound salicin, was used for thinning the blood and inhibits clot (2). Quinin, a bitter alkaloid extracted from chinchona bark, and artemisinin, which is extracted from sweet wormwood, were used in malaria therapy (3). Morphine, a white bitter substance obtained from opium, was used in medicine to reduce pain (4). Paclitaxel extracted from callus of the yew tree was used extensively in cancer treatment (5).

Polyphenols are important components of plant extracts as well as being consumed in food as dietary polyphenols. The bioactivities of polyphenols have been widely reported. According to Cragg et al., 30% of anti-inflammatory drugs developed in 1980s were derived from natural polyphenols (6). Oat polyphenols and ampelopsis grossed polyphenols were reported for their ability to clear freedom base such as 1,1-diphenyl-2-picrylhydrazyl radical 2,2-diphenyl-1-(2,4,6-trinitrophenyl) hydrazyl, superoxide anion radical, thus restraining cancer cells from developing, and enhancing immunity (7). In addition, kelp polyphenols exhibited bactericidal activities toward *Penicillium* and *Candida albicans* (8). Seeram et al. also found that anthocyanidin isolated from blueberries possess anti-cancerous bioactivities and induced apoptosis in human colon cancer cells (9). Moreover, cinnamon polyphenols were reported by Cao et al., for their ability to decrease blood glucose by regulating the glucose transporter gene expression in murine macrophages (10). Thus, a wide variety of phenolic substances possess various and striking properties.

When the human body is infected by bacteria or other pathogens, or injured from external or internal stimuli, a battle will begin between the immune system and external antigens. This process is known as inflammation. Hallmarks of inflammation include redness, warmth, swelling, and pain. Modern lifestyle is summed by inadequate daily physical activity, accompanied with excess caloric intake, causing energy surplus (11). During obesity, the enhanced level of triglyceride synthesis and break-down reactions in adipose tissue release excessive free fatty acids (FFAs), causing localized inflammation by recruiting immune cells including T cells and macrophages, thus contributing to lifestyle-related diseases such as atherosclerosis and non-alcoholic steatohepatitis (12, 13). During an inflammatory response, cyclooxygenase (COX)-2 catalyzes the conversion of arachidonic acid to prostaglandins (PGs) and thromboxane, including PGE_2_, which is a species of endogenous pain-producing substance (14). Meanwhile, COX-1, the other isoform of COX, acts as a house-keeping enzyme, appears to be responsible for the production of PGs that are important for homeostatic functions, such as maintaining the integrity of the gastric mucosa, mediating normal platelet function, and regulating renal blood flow (15, 16). The inhibition of COX-1 may induce gastrointestinal side effects.

Non-steroidal anti-inflammatory drugs (NSAIDs), such as aspirin, paracetamol, naproxen, celecoxib, etc., are major therapy for inflammatory pain. While effective in controlling pain, some NSAIDs are associated with significant side effects, most frequently gastrointestinal bleeding and cardiovascular events since they are non-selective COX-inhibitors, and the potential to combine with COX-1 may even cause complications that result in death. Therefore, COX-2-specific inhibitors (coxibs) are recommended by therapy guidelines to decrease NSAIDs-related side effects (17). In obese rats, administration of COX-2 by celecoxib, a COX-2 specific inhibitor, significantly reversed obesity-induced chronic inflammation and insulin resistance. COX-2 is demonstrated to play a pivotal role in chronic inflammation (18).

Taheebo polyphenols (TP) are water extracts of *Tabebuia* spp. (Bignoiaceae), taken from the inner bark of the *T. avellaneda* tree. This tree has been used extensively as folk medicine in Central and South America to treat diseases such as bacterial infections, cancer, and inflammation-related pain, while improving immune function through regulation of immune cell subtypes (19). Recently, two research teams have discussed the potential of TP in treating obesity or inflammation using animal experiments, both gained remarkable results while the underlying mechanism being not fully clarified (20, 21). The former study indicated the potential of ethanolic extracts of Taheebo on fatty liver and obesity treatment through regulation of related gene expression, whereas the latter demonstrated that TP might contribute to pain relief under various circumstances, such as thermally induced pain and acetic acid-induced pain. As the pharmacological activity of TP was discussed worldwide and raised concern for feasible and safe drug development, our study aimed to demonstrate the potential and mechanisms of TP as an anti-inflammatory substance without the side effects of COX-1 inhibition using an *in vitro* macrophage model, stimulated by FFA to mimic the *in vivo* adipose inflammation environment.

## Materials and Methods

### Preparation of TP

The dried inner bark of *Tabebuia avellanedae* (2.0 kg), which was generously provided by Taheebo Japan Co., Ltd. (Osaka, Japan) was extracted using with boiling water (12.8 L) two times for 1 h. The water solution was subjected to polyamide (400 g) column chromatography and eluted by water (6.0 L), 30% MeOH aq. (1.5 L), 40% MeOH aq. (1.5 L), 50% MeOH aq. (2.5 L), and 100% MeOH. The 50% MeOH aq. eluate was concentrated *in vacuo*. The residue (12.6 g) was followed by silicagel column chromatography and eluted by CHCl_3_–MeOH–H_2_O (14:6:1). Then, we used the concentrated fraction (7.7 g) which exhibited coloration in a dark green color by a mist of the ferric chloride reagent on thin-layer chromatography (Silica gel G) and is demonstrated to be polyphenols especially acteoside as the main constituent (22). The extracted TP were prepared to use after dissolved in culture medium and sterilization.

### Cell Culture

RAW 264.7 mouse macrophage cell line was cultured in high-glucose DMEM (Wako Pure Chemical Industries, Ltd., Osaka, Japan) supplemented with 10% fetal bovine serum (Biowest Ltd., Loire Valley, France), 100 U/mL penicillin, and 100 µg/mL streptomycin (Thermo Fisher, Rockford, IL, USA) at 37°C in a 5% humidified incubator with 5% CO_2_. Also, THP-1 human macrophage cell line was cultured as above except the fundamental culture was RPMI 1640 (Wako Pure Chemical Industries, Ltd., Osaka, Japan). RAW264.7 cells were stimulated with 500 µM FFA (Palmitic acid, Tokyo Chemical Industry Co., Ltd., Tokyo, Japan) prepared by being dissolved and assembled in 10% bovine serum albumin (Thermo Fisher, Rockford, IL, USA) in hot atmosphere (55–60°C), until fully dissolved. THP-1 cells were cultured in the presence of 100 ng/mL PMA for 72 h. Adherent macrophages were treated with serum-free RPMI 1640 for 24 h and then stimulated using FFA produced as above. To determine the specific action of FFA, BSA was used as vehicle in the control groups.

### Cytotoxicity Assay and Determination of Nitric Oxide (NO) or PGE_2_ Production

To ensure the safety of TP, cytotoxicity assay was performed using Cytotoxicity LDH Assay Kit (Dojindo Laboratories, Kumamoto, Japan) according to the manufacturer’s instructions. Cells were cultured in culture medium with or without TP at indicated concentrations (10–1,000 µg/mL), or FFA solely (0–500 µM), or combined, cultured for 24 h followed by LDH assay. Measurement of nitrite in medium was used as an indicator of NO production. Culture supernatants were collected and nitrite, the stable reaction product generated from NO with molecular oxygen, was measured using Griess reagent (Cayman Chemical Co., Ann Arbor, MI, USA) according to the manufacturer’s instructions. PGE_2_ production was measured with ELISA according to the manufacturer’s instructions (Cayman Chemical Co.).

### Real-time (RT) Quantitative Polymerase Chain Reaction (PCR)

To determine mRNA expression in cells, the cells were quickly harvested and stored at −80°C. Total RNA was extracted using RNeasy Mini Kit (Qiagen, Valencia, CA, USA) according to the manufacturer’s instructions and assessed for purity using the NanoDrop system (NanoDrop Technologies, Wilmington, DE, USA). Total mRNA was reverse transcribed to cDNA using High Capacity cDNA Reverse Transcription Kit (Applied Biosystems, USA) according to the manufacturer’s instructions. PCR was performed using the Fast 7500 RT-PCR system (Applied Biosystems) using Fast SYBR^®^ Green PCR Master Mix kits (Applied Biosystems). The thermal profiles consisted of 10 min at 95°C for denaturing, followed by 40 cycles of 95°C for 15 s, annealing at 60°C for 1 min. β*-Actin* or *glyceraldehyde-3-phosphate dehydrogenase* mRNA was used as the house-keeping gene for RAW 264.7 or THP-1 cells, respectively, and all data were represented relative to its expression (i.e., using standard curve methods) as fold change from the control group. Specific PCR primer pairs for each studied gene are shown in Table 1.

**Table 1:** Primer sequence.

A. Murine	
mRNA	Sequence (5′→3′)
*Ptgs-1 (COX-1)*	F:ATGAGTCGAAGGAGTCTCTCG
	R:GCACGGATAGTAACAACAGGGA
*Ptgs-2 (COX-2)*	F:TTCCAATCCATGTCAAAACCGT
	R:AGTCCGGGTACAGTCACACTT
*IL-1β*	F:GAAATGCCACCTTTTGACAGTG
	R:TGGATGCTCTCATCAGGACAG
*IL-6*	F:AACGATGATGCACTTGCAGA
	R:TGGTACTCCAGAAGACCAGAGG
*iNOS (Nos)*	F:GTTCTCAGCCCAACAATACAAGA
	R:GTGGACGGGTCGATGTCAC
*TNF-α*	F:CCTCCCTCTCATCAGTTCTA
	R:ACTTGGTGGTTTGCTACGAC
*β-actin*	F:TAAAGACCTCTATGCCAACACAGT
	R:CACGATGGAGGGGCCGGACTCAT
**B. Human**	
*PTGS-1 (COX-1)*	F:TGCCCAGCTCCTGGCCCGCCGCTT
	R:GTGCATCAACACAGGCGCCTCTTC
*PTGS-2 (COX-2)*	F:GGGCAAAGACTGCGAAGAAG
	R:CCCATGTGACGAAATGACTG
*GAPDH*	F:CGGAGTCAACGGATTTGGTCGTAT
	R:AGCCTTCTCCATGGTGGTGAAGAC

### COX-1 and COX-2 Activity Assay

Changes in the activities of COX-1 and COX-2 isoforms were measured with COX inhibitor screening assay kit (Cayman Chemical Co.). COX activity assay is a colorimetrical assay of the peroxidase component of COXs. The working principle is based on the monitoring of the competition between PGs and a PG-acetylcholinesterase conjugate (PG tracer) for a limited amount of PG antiserum. According to the manufacturer’s protocol, each reaction tube was set with the appropriated amount of assay buffer, heme, COX-1, or COX-2, and various concentration of TP. After the COX reaction, COX-derived PGs were directly measured by ELISA procedure. Arachidonic acid was used to initiate the reaction. After 18 h incubation at room temperature, the colorimetric substrate was added. The absorption was measured on a plate reader at 405 nm. All experiments were performed in triplicate.

### Computational Molecular Docking

The COX-1 or COX-2 enzyme is a homodimer in the crystalline form. Docking studies were conducted with the enzyme monomeric unit, and the enzyme active site was situated at the bottom along a narrow hydrophobic channel. Compound model were prepared by Chem Office. A molecular docking analysis of the peptides was performed using Autodock 4.2 (CCDC, UK^1^). To accelerate the docking process, the progress was performed through VcpPt. The docking modes were observed by Autodock tools (23). The COX-2 and iNOS crystal structure (Protein Data Bank ID:5JVY and 1NSI) were obtained online,^2^ and the protein structure was prepared using Accelrys Discovery Studio 4.0 software (Accelrys Software, Inc., San Diego, CA, USA). The energies of the peptides for the computational docking study were minimized by applying the CHARM22 force field using the Accelrys Discovery Studio 4.0 software. Gasteiger–Hückle charges were assigned to the enzyme after removing the water molecules and adding all hydrogen atoms. The ligand conformers were treated as flexible, and the protein structures were treated as rigid during the docking process. The best docking results were the conformation with the lowest binding free energy (24).

### Statistical Analysis

Experiments were performed in triplicate. Data analyses were performed using Prism 6.0. Data are expressed as mean ± SD and analyzed using one-way analysis of variance with Kruskal–Wallis with Dunn’s *post hoc* test performed in triplicate. Significant differences were set at *p* < 0.05.

## Results

### Effects of TP or FFA on Cell Viability of Murine and Human Macrophages

**Figure 1:**
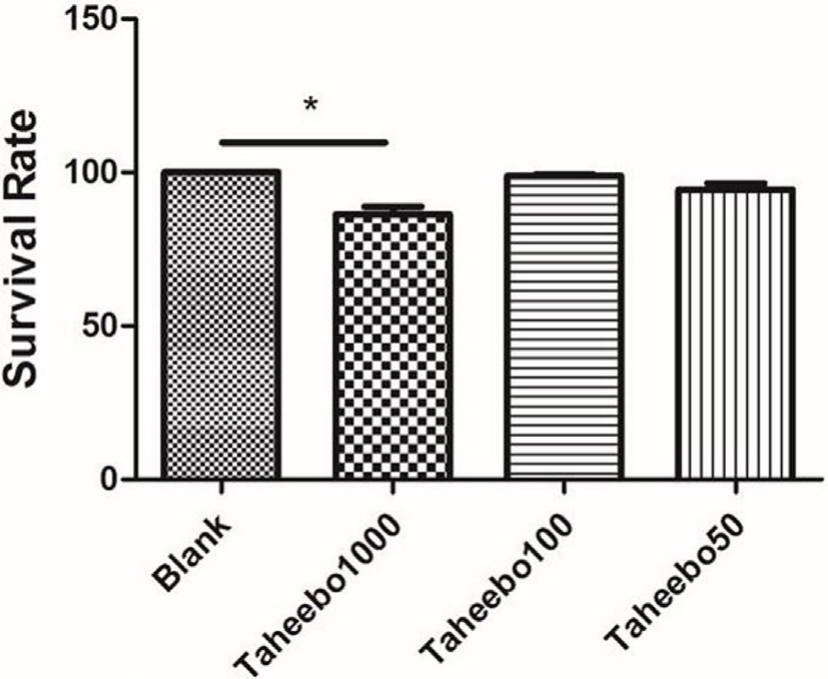
Effects of various concentration of Taheebo polyphenol and 500 µM free fatty acid on cell survival rates. *p < 0.05, significantly different compared with the blank condition.

To ensure the safety of TP and to verify that FFA did not induce changes in cell viability, LDH assay was performed. RAW 264.7 cells or THP-1 cells were cultured with or without TP at indicated concentrations (10–1,000 µg/mL), or FFA solely (0–500 µM), or combined, followed by LDH assay. TP did not affect the overall cell viability treated up to 100 µg/mL, nor did 500 µM FFA solely (Figure 1).

### Effects of TP on iNOS-Mediated NO and PGE_2_ Production of Murine and Human Macrophages

**Figure 2:**
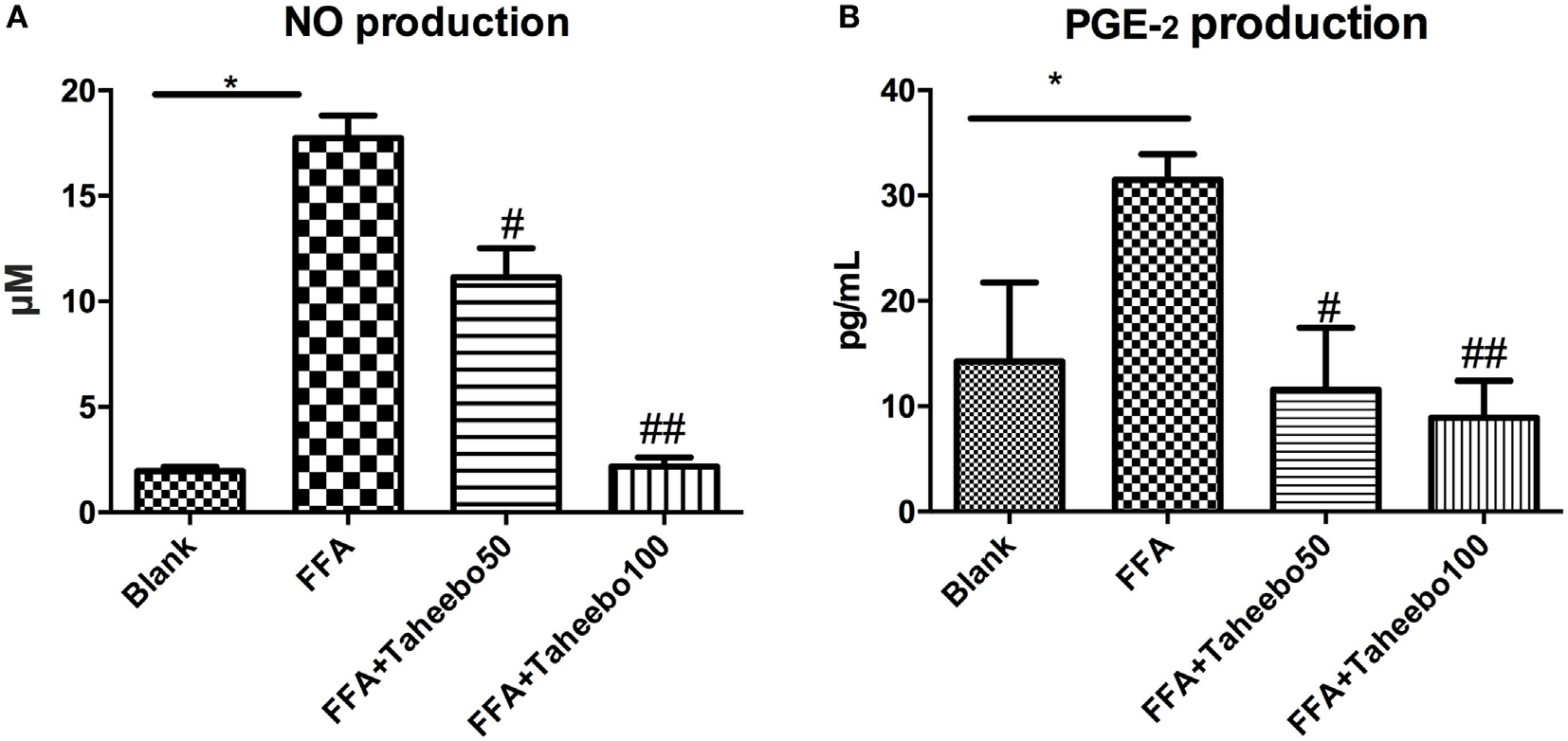
Effect of Taheebo polyphenol (TP) on expression of free fatty acid (FFA)-induced **(A)** nitric oxide (NO) production and **(B)** PGE_2_ production. RAW264.7 cells were treated with 50, 100 µg/mL or without TP, in the presence of absence of 500 µM palmitic acid for 24 h. Cell lysate was prepared and the concentrations of NO or PGE_2_ were measured by Griess assay or ELISA. Data are expressed as mean ± SD and analyzed using one-way analysis of variance with Kruskal–Wallis with Dunn’s *post hoc* test performed in triplicate. *p < 0.05, **p < 0.01, and ***p < 0.001, significantly different compared with the blank condition. ^#^*p* < 0.05, ^##^*p* < 0.01, and ^###^*p* < 0.001 significantly different compared with the FFA alone condition.

**Figure 3:**
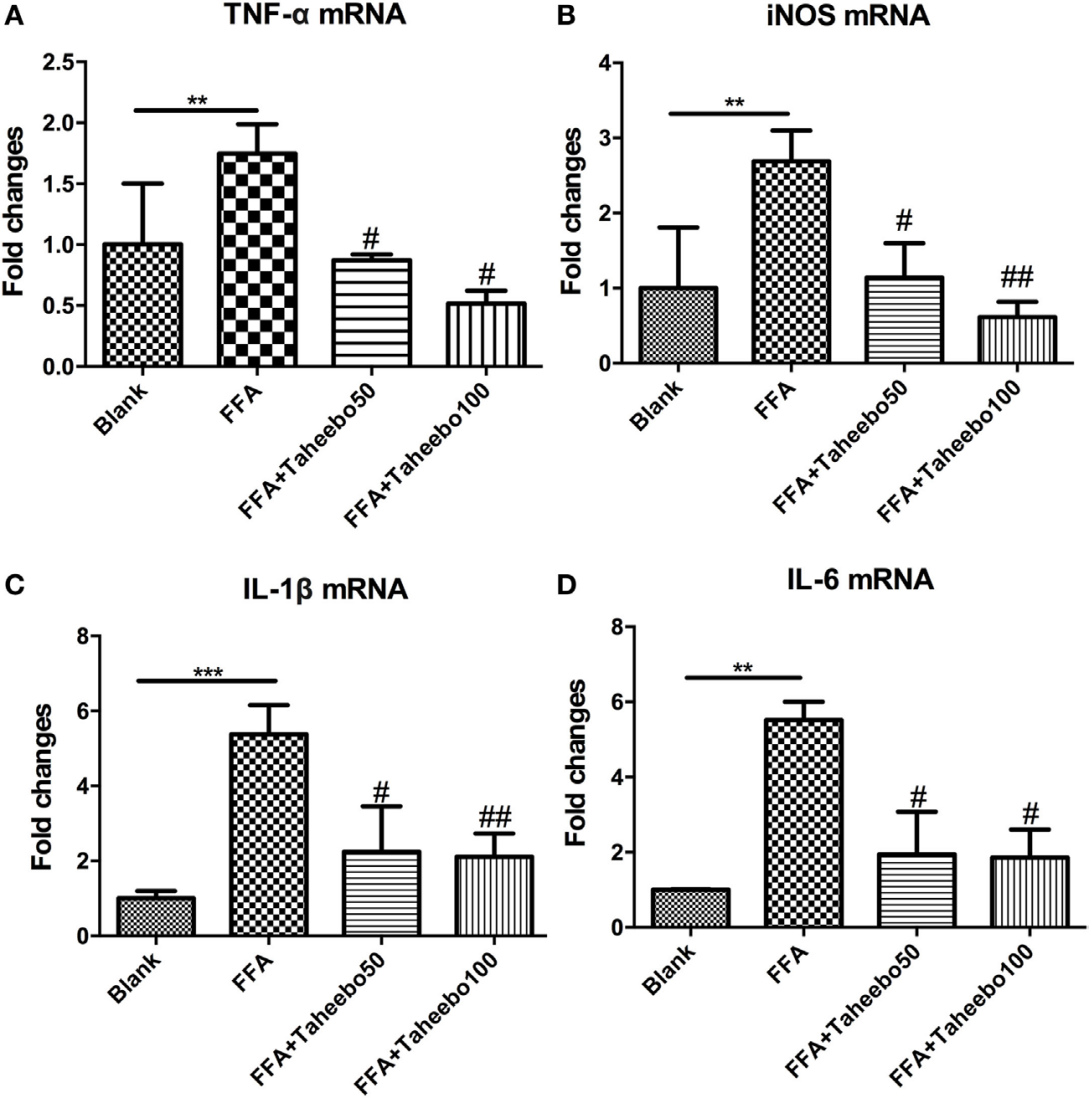
Effect of Taheebo polyphenol (TP) on expression of free fatty acid (FFA)-induced **(A)** TNF-α, **(B)** iNOS, **(C)** IL-1β, and **(D)** IL-6 mRNA (murine cell line). RAW264.7 cells were treated with 50, 100 µg/mL or without TP, with or without 500 µM palmitic acid for 24 h. Total RNA was prepared and expression levels of mRNA encoding TNF-α, iNOS, IL-1β, and IL-6 were measured by real-time polymerase chain reaction. β-actin was used as an internal control. Data are expressed as mean ± SD and analyzed using one-way analysis of variance with Kruskal–Wallis with Dunn’s *post hoc* test performed in triplicate. *p < 0.05, **p < 0.01, and ***p < 0.001, significantly different compared with the blank condition. ^#^*p* < 0.05, ^##^*p* < 0.01, and ^###^*p* < 0.001 significantly different compared with the FFA alone condition.

**Figure 4:**
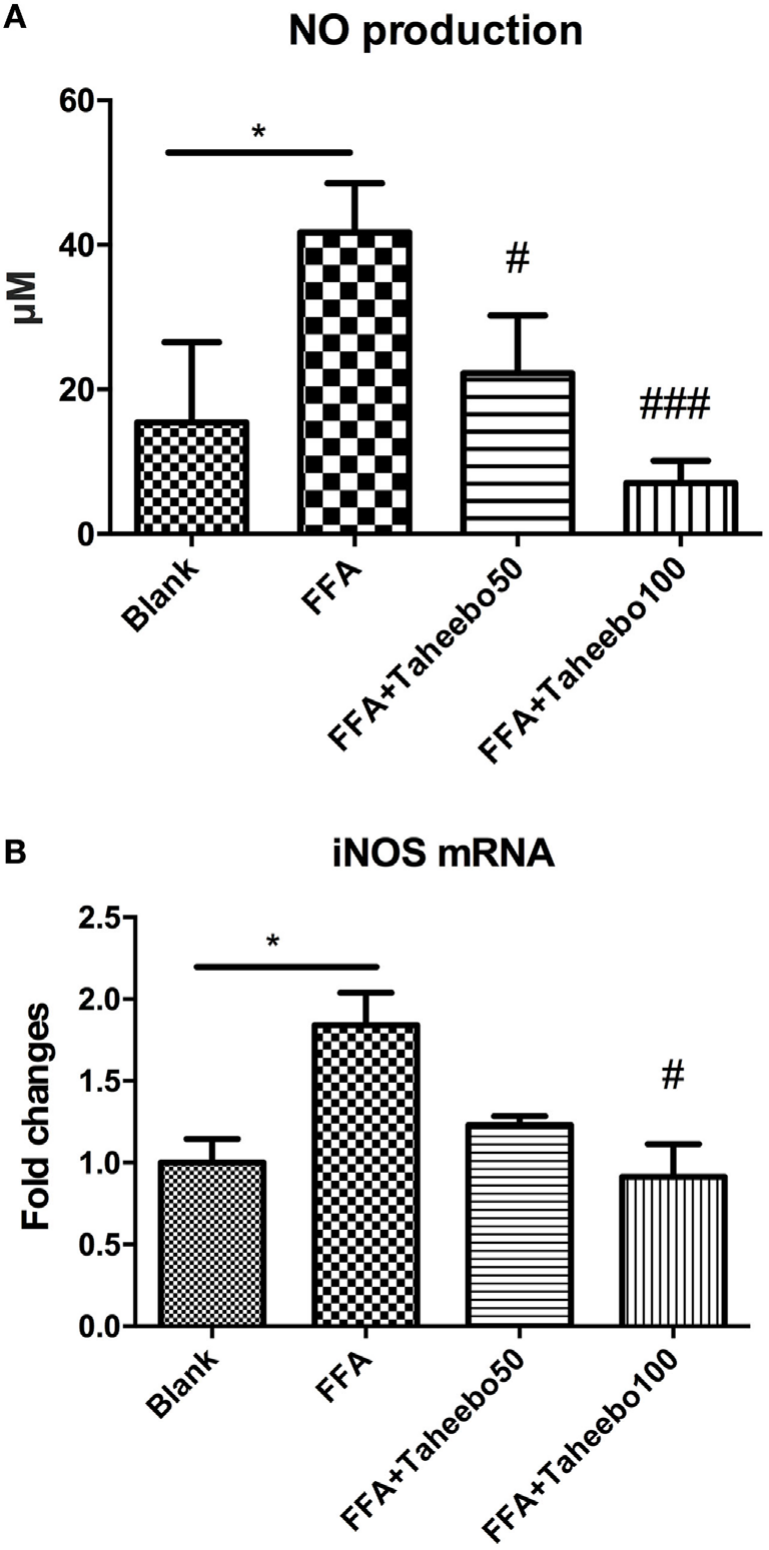
Effect of Taheebo polyphenol (TP) on expression of free fatty acid (FFA)-induced **(A)** nitric oxide (NO), **(B)** iNOS mRNA (human cell line). THP-1 cells were treated with 50, 100 µg/mL or without TP, with or without 500 µM palmitic acid for 24 h. Total RNA was prepared and expression levels of mRNA encoding iNOS was measured by real-time polymerase chain reaction. Glyceraldehyde-3-phosphate dehydrogenase was used as an internal control. Data are expressed as mean ± SD and analyzed using one-way analysis of variance with Kruskal–Wallis with Dunn’s *post hoc* test performed in triplicate. *p < 0.05, **p < 0.01, and ***p < 0.001, significantly different compared with the blank condition. ^#^*p* < 0.05, ^##^*p* < 0.01, and ^###^*p* < 0.001 significantly different compared with the FFA alone condition.

iNOS-mediated NO release is one of the major contributing factors during the early stages of inflammation. PGE_2_ is one of the crucial metabolites synthesized through the catalytic reaction mediated by COX-2 during the progressive stage of inflammation, mostly synthesized in substantial amounts of sites of inflammation. To investigate the effects of TP on NO and PGE_2_ production, Griess assay and ELISA were employed. As shown in Figures 2A,B and 3A, FFA-treated conditions significantly increased *Nos2* (murine iNOS mRNA), NO, or PGE_2_, whereas treatment of TP reversed the increase significantly in a concentration-dependent manner. As shown in Figure 4, up-regulated *NOS2* (human iNOS mRNA) expression and elevated NO production countered the decrease by TP in THP-1 human macrophage cell line.

### Effects of TP on Pro-inflammatory Cytokine mRNA Expression of Murine Macrophages

Pro-inflammatory cytokines such as TNF-α, IL-1β, and IL-6 play crucial roles in various inflammatory diseases. Since TP showed inhibitory activities toward inflammatory mediators NO and PGE_2_, we therefore investigated whether TP could regulate *NOS2, TNF-α, IL-1β*, and *IL-6* mRNA expression. As shown in Figure 3, the stimulation of FFA induced an increase in gene expression of each inflammatory mediator in murine cell lines. However, the increases were reduced by TP in a concentration-dependent manner.

### Effects of TP on *COX-1* and *COX-2* mRNA Expression of Murine and Human Macrophages

**Figure 5:**
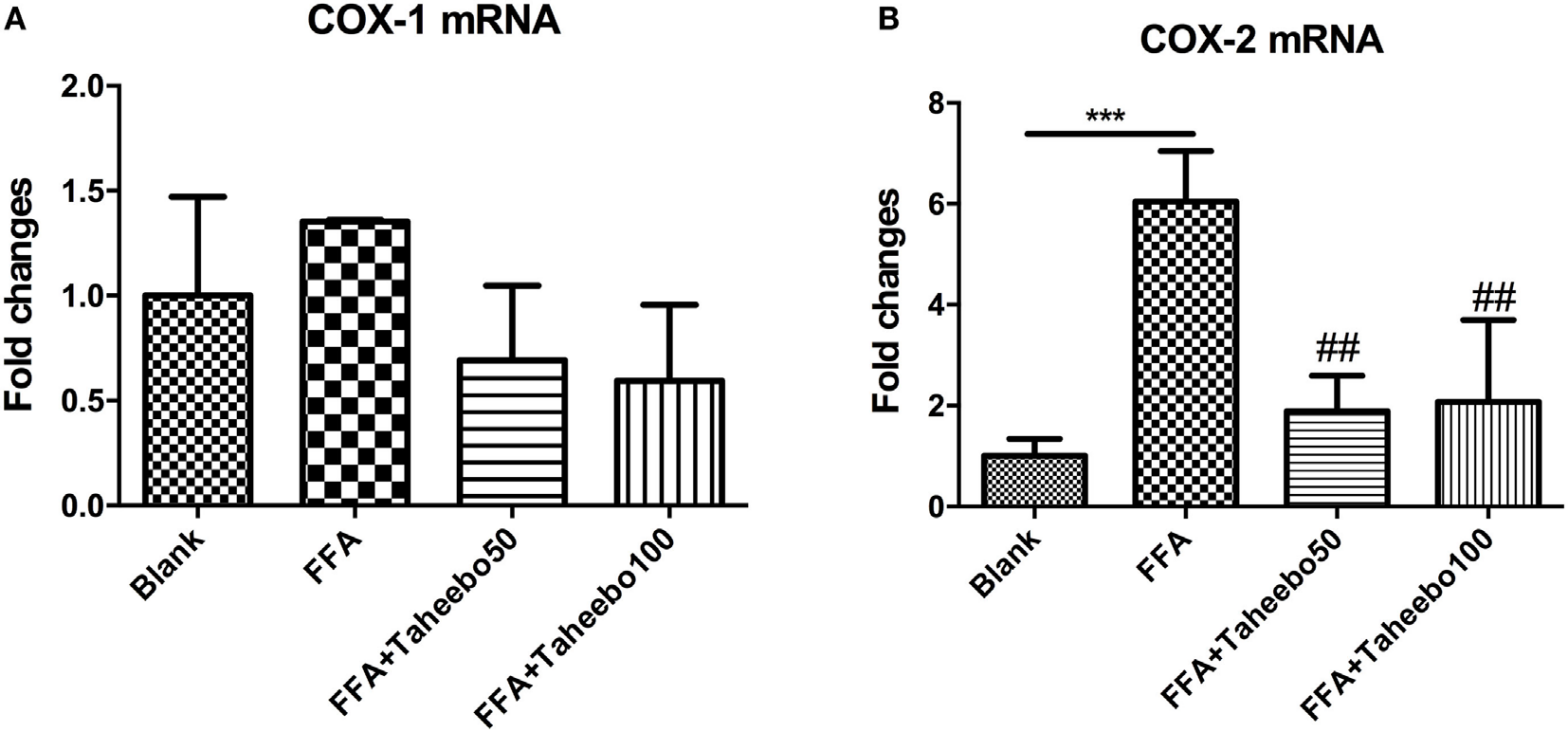
Effect of Taheebo polyphenol (TP) on expression of free fatty acid (FFA)-induced **(A)** cyclooxygenase (COX)-1 and **(B)** COX-2 mRNA (murine cell line). RAW264.7 cells were treated with 50, 100 µg/mL or without TP, with or without 500 µM palmitic acid for 24 h. Total RNA was prepared and expression levels of mRNA encoding COX-1 and COX-2 were measured by real-time polymerase chain reaction. Data are expressed as mean ± SD and analyzed using one-way analysis of variance with Kruskal–Wallis with Dunn’s *post hoc* test performed in triplicate. *p < 0.05, **p < 0.01, and ***p < 0.001, significantly different compared with the blank condition. ^#^*p* < 0.05, ^##^*p* < 0.01, and ^###^*p* < 0.001 significantly different compared with the FFA alone condition.

**Figure 6:**
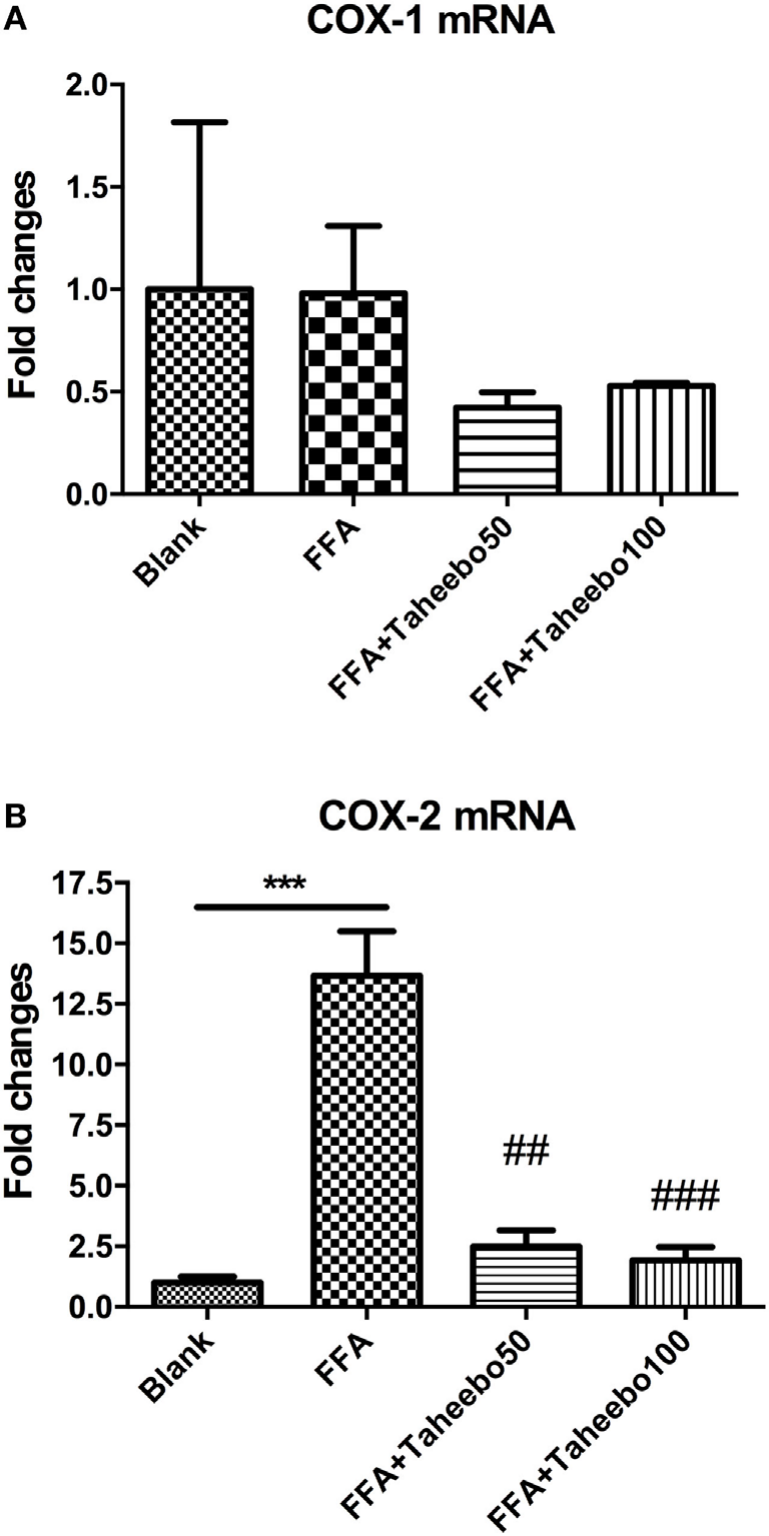
Effect of Taheebo polyphenol (TP) on expression of free fatty acid (FFA)-induced **(A)** cyclooxygenase (COX)-1 and **(B)** COX-2 mRNA (human cell line). THP-1 cells were treated with 50, 100 µg/mL or without TP, with or without 500 µM palmitic acid for 24 h. Total RNA was prepared and expression levels of mRNA encoding COX-1 and COX-2 were measured by real-time polymerase chain reaction. Glyceraldehyde-3-phosphate dehydrogenase was used as an internal control. Data are expressed as mean ± SD and analyzed using one-way analysis of variance with Kruskal–Wallis with Dunn’s *post hoc* test performed in triplicate. *p < 0.05, **p < 0.01, and ***p < 0.001, significantly different compared with the blank condition. ^#^*p* < 0.05, ^##^*p* < 0.01, and ^###^*p* < 0.001 significantly different compared with the FFA alone condition.

The enhanced production of NO and PGE_2_ during inflammation is generally attributed to the up-regulation of iNOS and COX-2, respectively. Therefore, we examined the effect of mRNA expression of *PTGS-2* (human COX-2 mRNA) *or Ptgs-2* (mice COX-2 mRNA) with or without TP treatment during stimulation of FFA on murine or human macrophage models. As shown in Figures 5 and 6, in both cell lines, FFA alone significantly induced targeted mRNA expression of both cell lines in a concentration-dependent fashion, TP reversed the mRNA up-regulation. But the significance of up-regulation on *PTGS-1* or *Ptgs-1* (human or murine COX-1 mRNA) expression was not observed.

### Effects of TP on COX Isoforms

**Figure 7:**
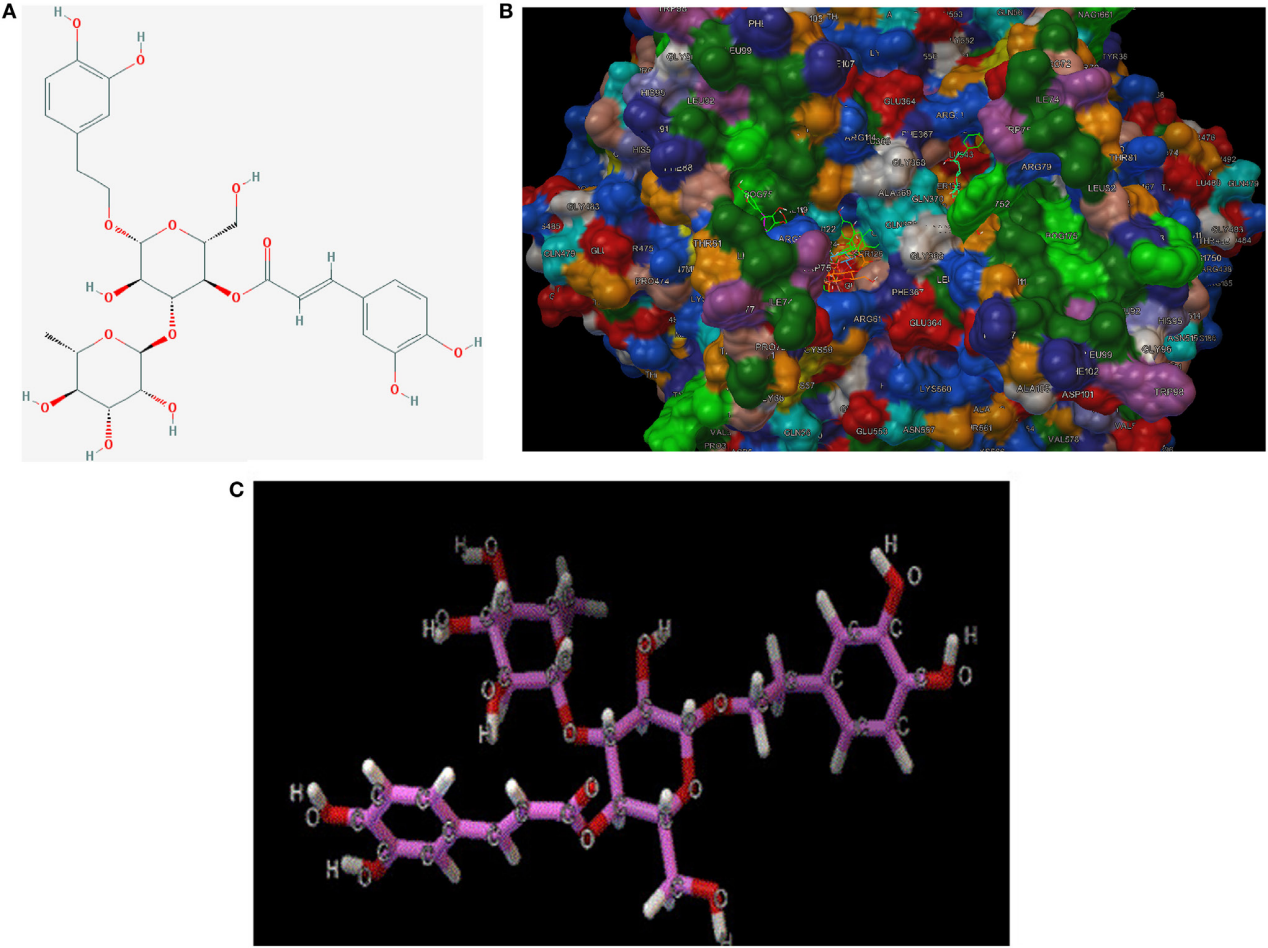
**(A)** 2D-structure of acteoside. **(B)** 3D-structure of acteoside, developed by Chemdraw Office 16.0. **(C)** Interaction between murine cyclooxygenase-2 protein, iNOS crystal and acteoside, performed by Autodock 4.0.

To make a tentative validation of whether and how TP could inhibit the catalytic activity of COXs, an *in silico* model using Autodock 4.0 was employed. The molecular structures of representatives of TP were drawn by ChemOffice 16.0. In the reference of the study of Suo et al., the representatives of TP were chosen to be acteoside [(22), Figure 7A]. As shown in Figure 7B, the catalytic center of COX-2 was occupied by representatives of TP. We also performed docking tests between iNOS and representatives of TP, similar to the interactions between TP representatives, the binding energy are at low levels and the catalytic center was occupied by each representative. The free energy released during this binding process was shown in Table 2.

**Table 2:** Binding energy between ligands and enzymes (best pose).

Ligand/binding energy	COX-2	iNOS
Acteoside	−9.3	−8.9

**Figure 8:**
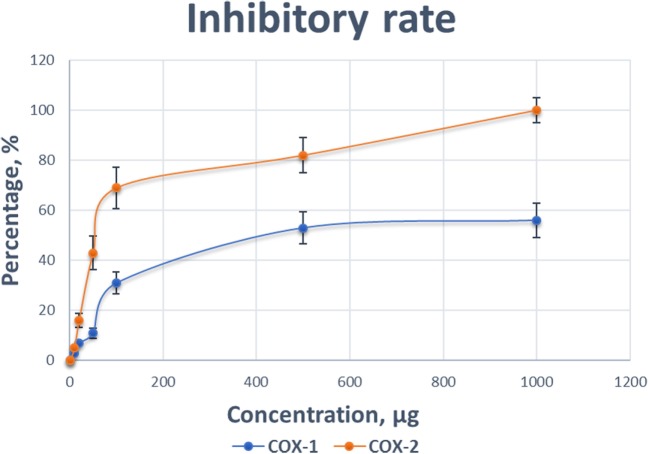
Comparison of the inhibitory effect of various concentrations of Taheebo polyphenols on the activity of cyclooxygenase (COX)-1 and COX-2. Experiments are performed in triplicate.

To support our hypothesis that TP might be a COX-2 selective inhibitor, we performed a COX inhibiting manner test using a commercial kit. As shown in Figure 8, the inhibitory effects of various concentrations of TP (0.001–1 mg/mL) on the activity of COX-1 and COX-2 isoforms. As both concentration-response curves were shown for comparison, the inhibitory effect of TP on the COX-2 enzyme system was significantly greater than on the COX-1 isoenzyme.

At concentration as low as 0.001 mg/mL, TP started to reduce the activity of the COXs. At the concentration of 1 mg/mL, the COX activity was completely inhibited. The preferred inhibitory effects of TP were apparent at concentrations between 0.1 mg/mL, where COX-1 was inhibited by 11%, while COX-2 was inhibited by 53%. At a concentration of 0.5 mg/mL, COX-1 was inhibited by 31%, while COX-2 was inhibited 82%. Table 3 shows the concentrations of TP required to inhibit the activities of COX-1 and COX-2 isoform by various inhibitory rates.

**Table 3:** Concentrations of Taheebo polyphenols required to achieve 50, 75, and 100% inhibition of cyclooxygenase (COX)-1 and COX-2 activities (milligram/milliliter).

Isoform	50%	75%	100%
COX-1	0.57 ± 0.05	0.76 ± 0.10	–
COX-2	0.12 ± 0.02	0.40 ± 0.02	0.88

## Discussion

The main bioactive components of TP have been reported recently. Awale et al. isolated 2 new iridoids and a new phenylethanoid glycoside and 12 known compounds from Brazilian *T. avellanedae*, which were indicated to possess NO production inhibiting effects in an LPS-activated J774.1 macrophage-like cell model. Acteoside is a well-studied phenylethanoid glycoside, showing various kinds of biological activities in many studies. Among those reported so far, the modulating activity of acteoside on NO production raised widespread interest (25, 26). However, neither the selectivity of TP nor acteoside on COX-1 and COX-2 has been reported.

Free fatty acid concentration is elevated in adipose tissue during obesity, thus causing activation of macrophages *via* various signaling cascades through FFA and toll-like receptor pro-inflammatory signal transduction, accompanying by increased infiltration of macrophages. Excessive FFA released from visceral adipose tissue is long considered to be associated with the pathogenesis of chronic inflammatory disease and metabolic syndrome, such as non-alcoholic steatohepatitis and type 2 diabetes. FFA has been shown to activate a pro-inflammatory profile in RAW 264.7 cells through transduction of toll-like receptors (27).

Recently, dietary polyphenols have become one of the most popular supplements worldwide and are believed to elicit anti-oxidant, anti-obesity, anti-diabetic, anti-carcinogenic, anti-inflammatory, and immunomodulatory properties (28). According to Yoon and Baek, the molecular targets of polyphenols with anti-inflammatory properties might be concluded as follows: (i) inhibition through the arachidonic acid-dependent pathway (AADP), during which COX and/or lipoxygenase, where hydroperonxyeicosateraenoic acids are synthesized and then secreted, or phospholipase A_2_; (ii) inhibition through arachidonic independent pathway (AAIP), during which peroxisome proliferator-activated receptors, nuclear transcription factor κB (NF-κB), *NSAID activated gene-1*, and/or nitric oxide synthase are activated (29, 30). The representative polyphenols exerting their actions through the former signal pathway are galangin and luteohin, presenting AA peroxidation-inhibiting activities. The extract of green tea enriched with catechin and epigallocatechin galate, is a kind of typical AADP and AAIP-inhibiting polyphenol, was well-known for its anti-inflammation properties, playing its significant roles through inhibiting the accumulation of PGE_2_, the classical metabolite of AADP, as well as inhibiting IL-6, and monocyte chemotactic protein-1, which are typical mediators of NF-κB, essential branch of AAIP. Our data showed that TP could regulate the downstream outcomes of NF-κB such as IL-1β, IL-6, TNF-α, and metabolites of iNOS and COX such as NO and PGE_2_, indicating that both classical pathways in which polyphenols exhibit their anti-inflammatory properties were blocked by TP. According to our results, the inhibitory effects of TP on FFA-induced NO and PGE_2_ production might be partly due to the suppression at the transcriptional level. Our data showed that TP presented COX selectivity, which allowed us to arrive at the hypothesis that TP might possess COX-2-specific inhibitory properties. This is supplementary to research conducted by other research teams using TP (31, 32).

To validate our presumption, we adopted molecular docking in our study. As a simulation technique, docking study provided an efficient method to perform initial screening between ligands, in our case, the representatives of TP and proteins (COXs and iNOS). Lower binding energy indicated that close combination may happen between representatives of TP and inflammatory enzymes. To make a double confirmation on our hypothesis, we carried out a COX inhibitor screening assay, adopting TP extracts.

Known as PG H Synthase or PGHS, COX is a bifunctional enzyme possessing both COX and peroxidase activities. This component converts a hydroperoxy endoperoxide (PGG_2_) from arachidonic acid, and this peroxidase component reduces the endoperoxide to the corresponding alcohol (PGH_2_), the precursor of PGs, thromboxanes, and prostacyclins. As COX-1 is constitutively expressed in a variety of cell types and is involved in normal cellular homeostasis, a variety of stimuli, such as phorbol esters, lipopolysaccharides, and cytokines, lead to the induced expression of a second isoform of COX, COX-2. COX-2 is responsible for the biosynthesis of PGs under acute inflammatory conditions. Side effects with the clinical use of PG synthase blockers are caused by the non-selectivity of inhibition on both COX-1 and COX-2 isoforms (33). To assess the selectivity of PG synthase inhibitor, we investigated the effect of TP on the activity of COX-1 and COX-2 isoforms. For TP, the concentration required to cause a 50% loss of COX-1 is much higher than COX-2. An approximate 50% loss of activity in COX-2 induced by TP coincides with a moderate loss of activity in COX-1 (11% loss of activity). In summary, TP showed both inhibitory effects on *COX-2* mRNA expression and protein level, may possess the double inhibitory in treatment of chronic inflammation-related diseases, as typical anti-inflammatory drugs do, for example, Indomethacin is extensively used as a kind of NSAIDs, but in researches conducted by Anderson et al. and Beer et al., respectively, Indomethacin is able to act on *COX-2* mRNA expression as well as exerting COX-selective activity (34, 35).

A study conducted recently employing 3T3-L1 adipose cell line showed that lipid end-product 4-Hydroxy-2-nonenal is responsible for COX-2 enhancement when the p38MAPK pathway is activated during inflammation (36). Considering the purported anti-oxidative properties of TP and macrophage infiltration during obesity, this new finding, which should have COX-2 regulating function, may further contribute to obesity-related chronic inflammation in adipose tissue (37).

Taken together, our results suggest that TP has a preference in COX-2 inhibition while exerting abilities on regulating *COX-2* mRNA expression, which may reduce the possibility of side effects by non-specific inhibitory behaviors during inflammation. We suggest that pre-clinical animal studies should be conducted to ensure these beneficial effects are adequate *in vivo*. The representative compound, acteoside from TP is considered to be particularly promising in treating lifestyle-related chronic inflammation.

## Conclusion

Our data suggest that TP is potentially useful in treating lifestyle-related chronic inflammation by attenuating inflammation caused by macrophage infiltration. The useful effects of TP are possibly due to the regulation of macrophages and the targeted inhibition of COX-2 activity, without affecting COX-1. The mechanism of this inhibition is probably due to acteoside, a key compound from TP, binding to the catalytic zone of COX-2, and the downstream catalytic activities thus attenuates PGE_2_ synthesis. TP are suggested to be one of the new generation of anti-inflammatory substances, exhibiting FFA-induced COX-2-selective inhibitory activities, and other anti-inflammatory actions.

## Ethics Statement

The protocol was approved by the Waseda University Animal Ethics Committee.

## Author Contributions

SM and KS designed research; SM, KY, and HL performed the experiment; SM and KY analyzed the data; SM, KS, YF, and AI wrote the paper. KS was the principal investigator and had primary responsibility for the final content. All authors read, critically revised, and approved the final manuscript.

## Conflict of Interest Statement

This work was supported by research funds endowed to KS from Taheebo Japan, Co., Ltd., Osaka, Japan.

## Footnotes

^1^http://www.ccdc.cam.ac.uk/products/csd/.

^2^http://www.rcsb.org/pdb.
