# Corrigendum: Taheebo Polyphenols Attenuate FFA-Induced Inflammation in Murine and Human Macrophage Cell Lines As Inhibitor of COX-2

**DOI:** 10.3389/fnut.2018.00002

**Published:** 2018-01-30

**Authors:** Sihui Ma, Koichi Yada, Hyunjin Lee, Youichi Fukuda, Akira Iida, Katsuhiko Suzuki

**Affiliations:** ^1^Graduate School of Sport Sciences, Waseda University, Shinjuku, Japan; ^2^Faculty of Sport Sciences, Waseda University, Shinjuku, Japan; ^3^Research Organization for Nano & Life Innovation, Waseda University, Shinjuku, Japan; ^4^Faculty of Agriculture, Kindai University, Higashi-osaka, Japan

**Keywords:** RAW264.7 cells, COX-2, THP-1 macrophages, free fatty acids, inflammation

In the article titled “Taheebo polyphenols attenuate FFA-induced inflammation in murine and human macrophage cell lines as inhibitor of COX-2” (1), there was an error regarding the protein PDB ID, which should be clarified as follows.

PDB ID for COX-2 protein used in the docking procedure is 5JVY (https://www.rcsb.org/pdb/explore/explore.do?structureId=5JVY), though in the article it was noted to be 4uxb.

The original article has been updated.

## Conflict of Interest Statement

The authors declare that the research was conducted in the absence of any commercial or financial relationships that could be construed as a potential conflict of interest.
